# Intramuscular Fat as a Biomarker of Muscle Health: Strengths, Limitations and Open Challenges

**DOI:** 10.1002/jcsm.70389

**Published:** 2026-09-30

**Authors:** Christopher Spence, Martin Alberto Belzunce, Anna Di Laura, Johann Henckel, Alister J. Hart

**Affiliations:** ^1^ Department of Orthopaedics and MSK Science, Division of Surgery and Interventional Science, Faculty of Medical Sciences University College London London UK; ^2^ Cleveland Clinic London London UK; ^3^ Royal National Orthopaedic Hospital Stanmore UK; ^4^ Centro Universitario de Imágenes Médicas (CEUNIM), Instituto de Ciencias Físicas (ICIFI UNSAM–CONICET), Escuela de Ciencia y Tecnología Universidad Nacional de San Martín San Martín Buenos Aires Argentina; ^5^ Department of Mechanical Engineering University College London London UK

**Keywords:** AI, biomarker, CT, fat infiltration, imaging modalities, intramuscular fat, MRI, MRS, muscle, ultrasound

## Abstract

Intramuscular fat (IMF), also known as myosteatosis, refers to the accumulation of lipids within and around skeletal muscle fibres. Excess IMF is increasingly recognised as a sign of poor muscle quality, linked to reductions in strength, power and mobility, and associated with adverse outcomes in metabolic disease, osteoarthritis, cardiovascular diseases and cancer. It is important to note that an increase in intramuscular fat is not always pathological. These associations and the ongoing uncertainty have sparked growing interest in IMF as a biomarker of muscle health beyond muscle mass and cross‐sectional area. This narrative review synthesises current evidence on IMF as a biomarker of muscle health, critically evaluating its definition, measurement, biological variability, responsiveness to intervention and ongoing challenges for clinical application. We first clarify terminology, distinguishing intramyocellular lipids, extramyocellular lipids, intermuscular adipose tissue and subcutaneous adipose tissue. Advances in imaging techniques are assessed: Magnetic resonance imaging and spectroscopy provide accurate compartmental lipid quantification; computed tomography offers practical, reproducible estimates of muscle density; and ultrasound presents an accessible, though operator‐dependent, alternative. Across populations, IMF shows considerable variability: Levels increase with age, differ by sex and ethnicity and have strong associations with adiposity and activity. Normative values are emerging but are lacking standardisation and population‐specific reference ranges. Interventional studies show that IMF is modifiable. Acute endurance exercise reduces intramyocellular lipid stores, while long‐term aerobic and resistance training prevent or lower fat infiltration but increase levels of intramyocellular lipids. Dietary restriction and bariatric surgery reliably decrease IMF but may also cause loss of lean mass without sufficient resistance training or protein intake. Pharmacological therapies, particularly glucagon‐like peptide‐1 receptor agonists such as liraglutide and semaglutide, have recently been shown to lower IMF while enhancing muscle quality, despite some overall muscle reduction. These findings support IMF as a flexible biomarker that indicates intervention success and correlates with functional outcomes including strength, gait speed and insulin sensitivity. Key barriers remain: lack of standardised imaging protocols, no universally defined pathological thresholds and the complex challenge of interpreting IMF across different age groups, sexes and ethnicities. Overcoming these obstacles through consensus definitions, prospective multi‐ethnic research and the integration of advanced imaging with functional outcomes will be vital. If achieved, IMF could become a clinically important biomarker, complementing mass and strength‐based measures to guide treatments, assess risk and track muscle health throughout life.

**Abbreviations**
^1^H‐MRS: proton magnetic resonance spectroscopy
^31^P‐MRS: phosphorus magnetic resonance spectroscopy
AI: artificial Intelligence
ATP: adenosine triphosphate
BMI: body mass index
CKD: chronic kidney disease
CNN: convolutional neural network
CSA: cross‐sectional area
CT: computed tomography
DAG: diacylglycerol
DTI: diffusion tensor imaging
EMCL: extramyocellular lipids
EPA: eicosapentaenoic acid
GLP‐1 RA: glucagon‐like peptide‐1 receptor agonist
GMAX: gluteus maximus
HU: Hounsfield units
IMAT: intermuscular adipose tissue
IMCL: intramyocellular lipids
IMF: intramuscular fat
IMTG: intramyocellular triglycerides/intramuscular triglycerides/intramuscular triacylglycerols
IntraMAT: intramuscular adipose tissue
MASLD: metabolic dysfunction‐associated steatotic liver disease
MRI: magnetic resonance imaging
MRS: magnetic resonance spectroscopy
MSK: musculoskeletal
NIH: National Institutes of Health
PDFF: proton density fat fraction
QUS: quantitative ultrasound
SARCUS: SARCopenia through UltraSound
SAT: subcutaneous adipose tissue
TEs: echo times
US: ultrasound

## 1 Introduction

Intramuscular fat (IMF) refers to fat deposits within skeletal muscle, as either intramyocellular droplets or extramyocellular fat between fibres. Often called myosteatosis, it is a recognised feature of muscle ageing and disuse and is strongly associated with reduced muscle strength, power, and mobility [1]. Excessive IMF reflects impaired muscle quality, making it a promising biomarker for musculoskeletal (MSK) health.

Higher IMF content has been linked not only to sarcopenia in the elderly but also to a range of chronic conditions. It is associated with coronary microvascular dysfunction and independently predicts adverse cardiovascular outcomes [2]. It is closely linked to insulin resistance and type 2 diabetes, with intermuscular adipose tissue shown to impair muscle tissue insulin sensitivity [3]. In metabolic dysfunction‐associated steatotic liver disease (MASLD), greater IMF correlates with disease severity, visceral adiposity and gut dysbiosis [4]. Worsening muscle quality is similarly associated with increased mortality in chronic kidney disease (CKD) and poorer outcomes in cirrhosis, including post‐transplant complications [5, 6, 7, 8]. In oncology, myosteatosis has emerged as an independent prognostic marker across multiple malignancies [9, 10]. These associations illustrate the breadth of clinical contexts in which IMF demonstrated prognostic value and underscore the need for reliable, reproducible quantification.

Myosteatosis is observed in many states of muscle dysfunction. Ageing individuals often exhibit fatty infiltration in the thigh and calf muscles, which correlates with poorer balance and increased falls risk [11, 12, 13, 14]. Patients with hip osteoarthritis show gluteal muscle fat infiltration that qualitatively parallels disease severity [15, 16]. In neuromuscular disorders like muscular dystrophies, magnetic resonance imaging (MRI) fat fraction mapping has become an invaluable outcome measure to track disease progression, given the replacement of muscle by fat over time [17]. In otherwise healthy adults, sedentary behaviour or immobilisation quickly increases IMF content, while regular physical activity helps maintain lean muscle mass [15, 18, 19]. These trends emphasise IMF as a biomarker for muscle health, extending beyond merely volume measures. While ageing may lead to 5%–10% muscle loss over a decade, intramuscular fat fraction can double over the same period, better explaining functional decline [1, 13, 20]. IMF, therefore, complements traditional metrics such as muscle mass or cross‐sectional area.

However, translating IMF from research measurement to a standard clinical biomarker faces challenges. How should it be quantified?—Dixon MRI, computerised tomography (CT) or magnetic resonance spectroscopy (MRS) [21, 22]. What influences these measures, and what thresholds define pathology or predict outcomes?

This review addresses these questions by first clarifying terminology and precisely defining the various fat compartments within and around muscle. We will evaluate the strengths and limitations of current imaging techniques and consider the evidence on whether interventions can modify IMF and if its change signifies meaningful improvements in health. Finally, we outline unresolved challenges that must be addressed before IMF can be widely adopted as a biomarker in clinical practise.

## 2 Defining Intramuscular Fat

Intramuscular fat accumulation has been described using overlapping terms reflecting differences in anatomical scale, biological interpretation and measurement methods. Originating from histology, metabolism, imaging and clinical research, these terms are often used inconsistently to describe related but distinct lipid depots within skeletal muscle. To address this inconsistency, the National Institute on Aging organised a multidisciplinary workshop emphasising the need for clearer, standardised terminology [1]. The term myosteatosis is commonly used as an umbrella term for several distinct lipid depots, as summarised in Table 1 and Figures 1 and 2.

**TABLE 1:** Terminologies across the literature for myosteatosis summarised and compartmentalised for this review.

Category	Terms	Definition	How it is measured
Myosteatosis	Muscular fat infiltration Ectopic fat depot	An umbrella term encompassing all muscle fat infiltration, including IMCL, EMCL, intramuscular fat and intermuscular adipose tissue.	Not a single measurable compartment
Intramyocellular lipids (IMCL)	Intramyocellular lipids (IMCL) Intramyocellular triglycerides (IMTG) Intramuscular triglycerides Intramuscular triacylglycerols (IMTG)	Lipid droplets within muscle fibres serving as a significant metabolic store; IMCL encompasses all lipid species in muscle cells, with IMTGs being the most prevalent.	^1^H‐magnetic resonance spectroscopy (^1^H‐MRS) Biopsy
Extramyocellular lipids (EMCL)	Extramyocellular lipids (EMCL)	Lipids located in the extracellular space between muscle fibres and fascicles; defined histologically and discussed in studies using MRS or biopsy.	^1^H‐MRS, as a separate spectral peak from IMCL Biopsy
Intramuscular fat (IMF)	Intramuscular adipose tissue (IntraMAT) Interstitial fat Interstitial adipose tissue	A broad term commonly used in imaging studies to refer to fat signal or attenuation within muscle, which may reflect combined contributions from IMCL, EMCL and/or IMAT, depending on modality and analysis approach.	MRI (T1/T2/Dixon) CT (Hounsfield units/visual) US echo‐intensity
Intermuscular adipose tissue (IMAT)	Intermuscular adipose tissue/fat (InterMAT)	Adipose tissue located between different muscles beneath the deep fascia; directly visible on cross‐sectional imaging.	MRI (T1/T2/Dixon) CT (Hounsfield units/visual) US
Subcutaneous adipose tissue (SAT)		Adipose tissue located beneath the skin, superficial to the muscle fascia.	MRI (T1/T2/Dixon) CT US

**FIGURE 1:**
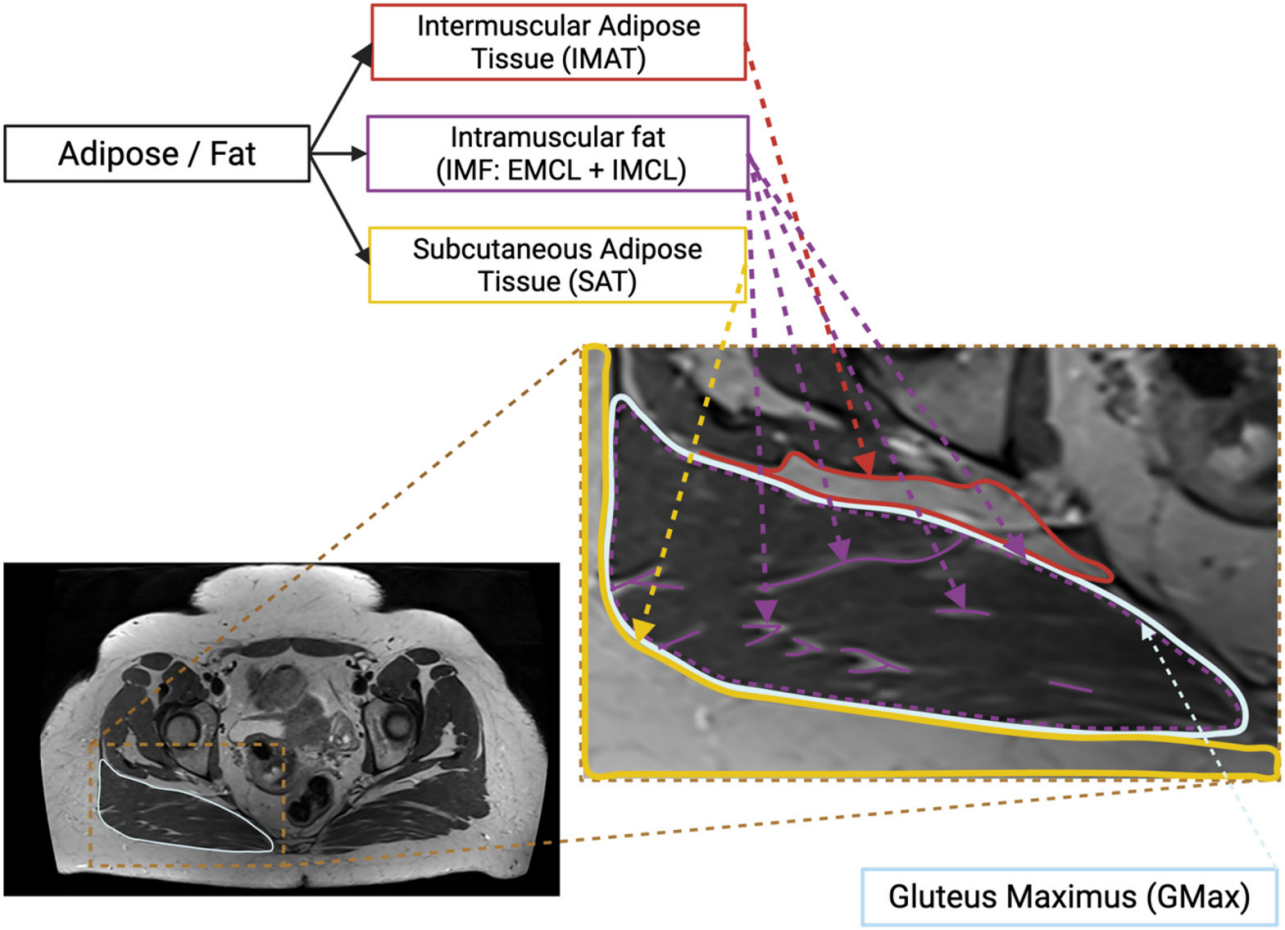
Adipose tissue compartments on a T1‐weighted magnetic resonance (MR) image of the pelvis. The schematic divides adipose tissue into intermuscular adipose tissue (IMAT), intramuscular fat (IMF) and subcutaneous adipose tissue (SAT); IMF comprises extramyocellular lipids (EMCL) and intramyocellular lipids (IMCL). On the magnified gluteus maximus (GMax, light blue outline), coloured arrows link each label to its location: yellow, SAT superficial to the muscle; red, IMAT separating GMax from the adjacent muscle; purple, IMF within the muscle. Solid purple lines mark visible interstitial (extramyocellular) fat. The dotted purple border indicates that microscopic EMCL and IMCL are present throughout the muscle but are not resolved in this image; 1H‐MR spectroscopy is required to distinguish them. *Source:* Created in BioRender. Spence, C. (2026) https://BioRender.com/951a00v.

**FIGURE 2:**
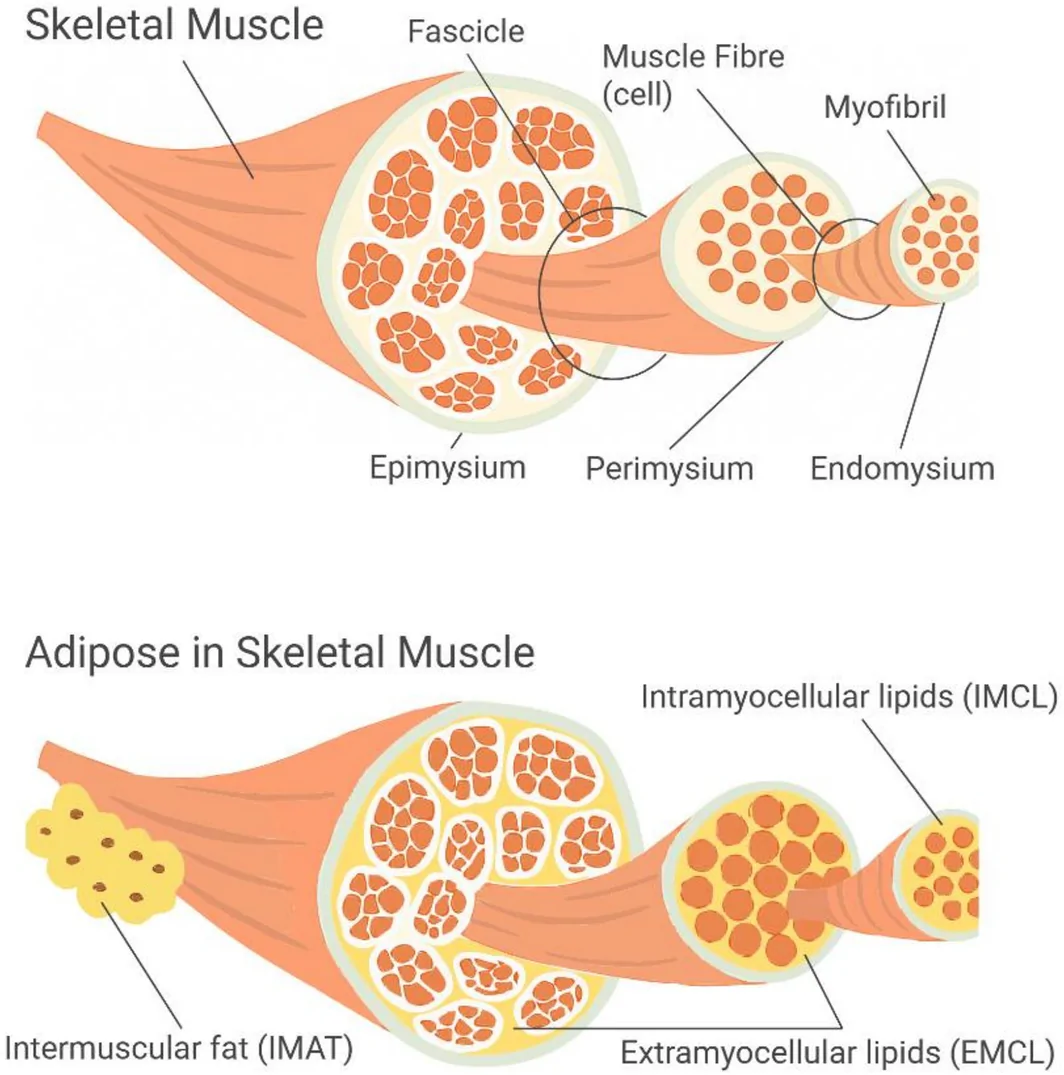
Skeletal muscle architecture and its associated fat compartments. The upper panel (Skeletal Muscle) shows the hierarchical organisation of skeletal muscle, from whole muscle to fascicle, fibre and myofibril, with the surrounding connective tissue layers (epimysium, perimysium, endomysium). The lower panel (Adipose in Skeletal Muscle) shows the fat depots that together contribute to intramuscular fat (IMF). Intermuscular adipose tissue (IMAT) lies between muscles beneath the deep fascia; extramyocellular lipids (EMCL) occupy the space between fibres and fascicles; and intramyocellular lipids (IMCL) are stored within the fibres. The relative contribution of each to measured IMF depends on imaging modality and resolution. *Source:* Created in BioRender. Spence, C. (2026) https://BioRender.com/7viz7s2.

At the microscopic level, intramyocellular lipids (IMCL) are stored as lipid droplets within muscle fibres and consist of various lipid species. The main component is the intramyocellular triglycerides (IMTG), also known as intramuscular triglycerides or triacylglycerols, which serve as an important, readily mobilised energy reserve [23, 24]. Other intramyocellular lipid species, particularly diacylglycerols (DAGs) and ceramides, are metabolically bioactive and play key roles in intracellular signalling pathways. When the accumulation of these lipid species exceeds the storage capacity of muscle fibres, known as lipotoxicity, insulin signalling is impaired and inflammatory pathways are activated [25]. Since IMTGs, DAGs and ceramides all have different intracellular responses, this suggests that intramuscular lipid composition, not only quantity, will be important when interpreting intramuscular fat as a biomarker [26, 27].

Extramyocellular lipids (EMCL) occupy the extracellular space between muscle fibres and fascicles. Microscopic EMCL is inferred rather than directly visualised, whereas larger interstitial collections are visible [1, 22, 28]. In imaging studies, intramuscular fat (IMF) and intramuscular adipose tissue (IntraMAT) refer to the lipid‐derived signal detected within muscle. This signal may reflect contributions from IMCL, EMCL and potentially intermuscular adipose tissue (IMAT), which lies between muscle groups beneath the deep fascia and is directly visible on MRI.

Macroscopic lipid stores near muscle, such as subcutaneous adipose tissue (SAT), are also clinically significant. SAT is located just beneath the skin, is visible on imaging and is distinct from muscle structures.

## 3 Imaging Methods Used to Evaluate Intramuscular Fat

Accurately quantifying intramuscular fat is challenging. Reliable assessments that differentiate and measure lipid compartments across multiple muscles in vivo are crucial for understanding myosteatosis as a muscle health biomarker. The most commonly used techniques are MRI and MRS, computed tomography (CT) and ultrasound (US), each with distinct capabilities. A critical appraisal of these methodologies is given below and summarised in Table 2.

**TABLE 2:** Summarising the key imaging modalities available for IMF assessment, highlighting their respective strengths, limitations and typical applications.

Modality	Strengths	Limitations	Typical use cases
Qualitative MRI (e.g., Goutallier/quartile scales)	Used in orthopaedics; simple, low post‐processing demand, good for gross muscle degeneration	Subjective, inter‐observer variability, limited sensitivity to small changes	Clinical decision‐making (e.g., surgical planning, rotator cuff repair)
Quantitative MRI (Dixon/PDFF)	High reproducibility; validated against MRS and histology; generates fat fraction maps of the whole muscle; suitable for longitudinal monitoring	Requires multi‐echo acquisition for accuracy; cost and accessibility limit routine use	Research trials in sarcopenia, type‐2 diabetes mellitus, cachexia
Magnetic resonance spectroscopy (MRS)	Gold standard for biochemical specificity; distinguishes IMCL vs. EMCL; non‐invasive metabolic profiling	Low spatial resolution, issues with voxel placement, poor reproducibility, not practical for whole muscle assessment	Mechanistic research (athlete's paradox, sarcopenia, metabolic studies)
Computerised tomography (CT)	Widely available; rapid; can quantify IMF via Hounsfield units; good reproducibility	Ionising radiation, less tissue specificity than MRI, cannot separate lipid compartments, density‐based assessment susceptible to fluid retention and glycogen variation	Opportunistic IMF assessment in clinical scans; population studies where CTs have already been acquired
Ultrasound (US/QUS)	Portable, inexpensive, bedside; allows assessment of architecture (fascicle length, pennation angle); QUS correlates with MRI estimates	Operator‐ and machine‐dependent; cannot resolve compartments; poor for deep muscles	Screening tool in sarcopenia/frailty; bedside monitoring in geriatric or rehabilitation settings

### 3.1 Magnetic Resonance Imaging

MRI is a powerful tool for visualising soft tissues and can directly quantify fat content using specialised sequences. It offers a spectrum of approaches from qualitative visual grading to advanced quantitative mapping and spectroscopy. MRI with standard clinical scanning protocols, including spin‐echo T1‐ and T2‐weighted sequences, allows for the qualitative and quantitative assessment of IMAT.

Qualitative MRI assessments remain widely used in clinical orthopaedics and sports medicine because of their simplicity, availability and established clinical relevance [29, 30]. Visual inspection of muscle signal and atrophy allows classification using systems such as the Goutallier scale and quartile grading [29, 30]. The Goutallier scale, for example, grades IMF from grade 0: normal muscle with no fat to grade 4: more fat infiltration than muscle. This offers clinicians a practical way to evaluate muscle degeneration, particularly in orthopaedic conditions [31]. These systems are practical and validated for routine clinical decision‐making, particularly in surgical planning, but are inherently subjective. Significant inter‐ and intra‐observer variability and lack of standardised acquisition parameters limit their reproducibility and sensitivity to subtle change, prompting the development of more objective methods [29, 32, 33].

### 3.2 Quantitative MRI Techniques

Within MRI, a range of quantitative techniques have been developed to characterise muscle composition beyond qualitative signal changes. Dixon‐based quantification exploits the chemical shift difference between water and fat protons to generate water‐only and fat‐only images [34, 35]. While two‐point acquisitions use two echo times (TEs), multi‐echo Dixon sequences acquire additional echoes to correct for field inhomogeneities, relaxation effects, spectral complexity and noise, substantially improving fat fraction accuracy [36, 37, 38, 39, 40]. Multi‐echo Dixon‐based techniques are recommended for accurate quantification of fat fraction, as they are least affected by these confounding factors.

The proton density fat fraction (PDFF) is calculated from these combined signals. PDFF represents the ratio of mobile triglyceride protons to total mobile protons (fat and water), providing a standardised measure of tissue fat fraction [41, 42]. This technique is well‐established in the research literature, with high levels of accuracy validated against MRS [43, 44], and is increasingly applied in clinical trials for conditions such as musculoskeletal and metabolic diseases [15, 45, 46, 47].

Despite these strengths, Dixon techniques are not without important limitations when applied to skeletal muscle. Many vendor‐implemented Dixon protocols were originally optimised for hepatic fat quantification and do not fully account for the more heterogeneous microstructure and lipid composition of muscle [22].

In addition to Dixon, other quantitative MR imaging methods, such as T2 mapping and Diffusion Tensor Imaging (DTI), have evolved. T2 mapping is highly sensitive to muscle oedema, inflammation and subtle changes in muscle hydration, providing additional information regarding muscle inflammation and trauma in conjunction with PDFF measures [48, 49]. Conversely, DTI assesses muscle microstructure based on the anisotropy of water molecule diffusion, offering a non‐invasive means of characterising muscle fibre integrity, orientation and architectural adaptations related to muscle atrophy and injury [50, 51]. Figure 3 demonstrates DTI for muscle assessment [52]. Although currently limited to research, combining these MRI imaging protocols with PDFF could generate detailed multiparametric profiles of muscle health, simultaneously assessing fat infiltration, inflammation and microstructural integrity.

**FIGURE 3:**
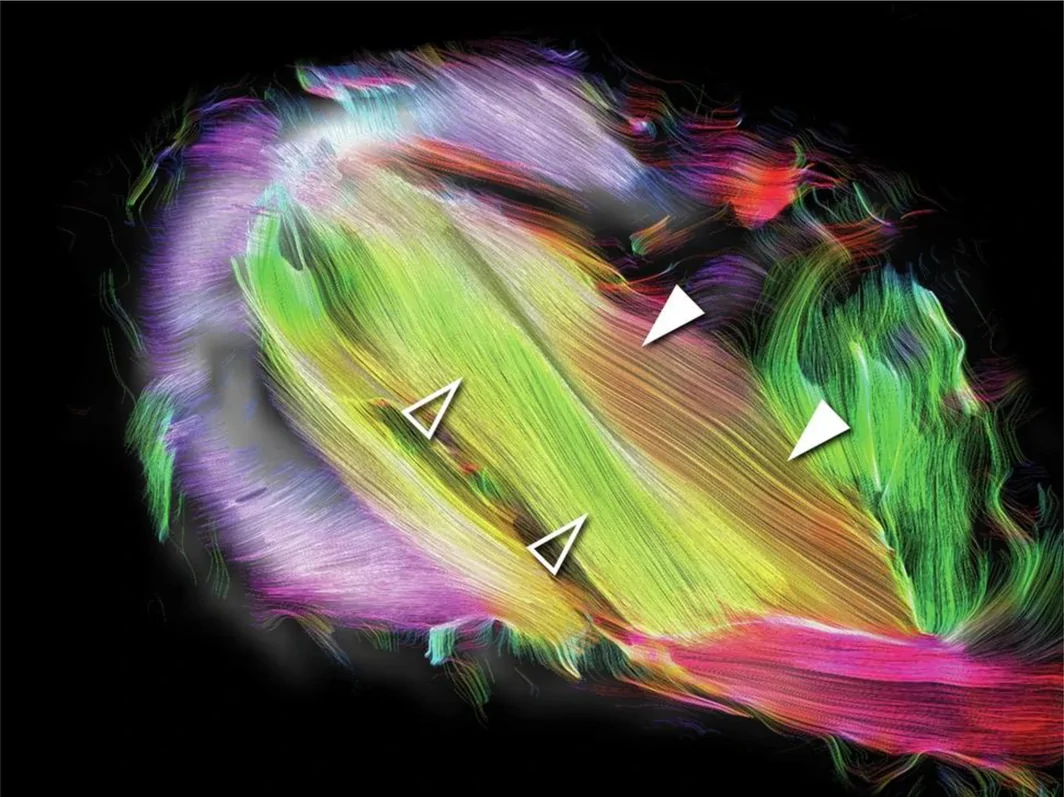
Axial diffusion tensor imaging slice with colour‐coded tractography of the right shoulder of a healthy 37‐year‐old male. Streamline colour encodes the principal direction of fibre diffusion: green, anteroposterior; red, mediolateral; blue, superoinferior. Solid arrowheads indicate the bipennate anterior portion of the supraspinatus muscle; the solid and outline arrowheads together mark the differing configurations of fibre bundles adjacent to the tendon. The figure was reprinted from a previously published open‐access journal article by Marth et al. under the terms of the Creative Commons CC BY license [52].

### 3.3 Magnetic Resonance Spectroscopy

MRS is a specialised MRI‐based technique that enables non‐invasive measurement of metabolites in skeletal muscle. Unlike conventional MRI, which characterises tissue structure, MRS provides biochemical information by exploiting differences in resonance frequencies of specific metabolites. Proton MRS (^1^H‐MRS) is most commonly used to quantify IMCL and EMCL, allowing direct distinction between these compartments based on their chemical shifts [28, 53]. This distinction is biologically relevant, as IMCL represents lipids stored within myocytes and is closely linked to metabolic flexibility, whereas EMCL reflects external lipid deposition [1, 54].

While ^1^H‐MRS is the most established approach for lipid quantification, other nuclei can be interrogated to probe complementary aspects of muscle metabolism. Phosphorus MRS (^31^P‐MRS) enables assessment of high‐energy phosphate metabolism, including phosphocreatine, adenosine triphosphate (ATP) and intracellular pH, providing complementary insight into mitochondrial function and muscle energetics [55]. These approaches are valuable in metabolic disease, sarcopenia and neuromuscular disorders, where alterations in energy metabolism may precede structural changes [56, 57, 58].

Despite its high biochemical specificity, MRS is limited by low spatial resolution, voxel placement variability and labour‐intensive acquisition, restricting reproducibility and feasibility for whole muscle or longitudinal assessment [22]. Although MRS has been used to monitor treatment effects, changes are often small, and IMCL has not yet been standardised as a clinical biomarker [59, 60]. As such, MRS remains best suited to mechanistic and metabolic research rather than routine assessment of muscle fat infiltration.

In summary, MRI and MRS both offer precision in IMF assessment. Qualitative methods provide clinically meaningful but subjective grading, quantitative Dixon‐based PDFF offers robust and validated measurement of muscular triglycerides, and MRS uniquely resolves lipid compartments and in vivo energy metabolism. The main barriers to broader adoption remain cost, accessibility and the need for standardised acquisition and analysis protocols. These are critical steps if IMF is to be utilised as a reliable biomarker across multi‐centre research and clinical settings.

### 3.4 Computed Tomography

CT has long been used to assess body composition, including muscle mass and fat infiltration. CT does not directly measure fat content but instead infers it from tissue radiographic density, expressed in Hounsfield units (HU). The default calibration for CT scanners is for water as the baseline, with a value of 0. Common HU values are −1000 for air (black on the grey scale), lean muscle tissue is typically much higher, ranging from +50 to +60 HU, and cortical bone is over +1000 HU [61]. As muscle tissue accumulates more fat infiltration, its average density decreases, and muscle with significant fatty infiltration appears less dense on CT scans [21].

CT assessment of IMF involves measuring mean attenuation in muscle regions or by calculating the cross‐sectional area (CSA) of muscle that falls below a certain HU threshold. Assessing the abdominal L3 vertebral level is a standardised approach widely used in body composition research and clinical studies. The skeletal muscle in this region is segmented, and the mean muscle attenuation is computed as an index of myosteatosis. CT attenuation thresholds commonly employed in oncology research include mean density values of less than 41 HU in individuals with a body mass index (BMI) under 25 kg/m^2^ or less than 33 HU in those with a BMI greater than or equal to 25 kg/m^2^ [10, 62].

Several clinical studies have confirmed the link between CT muscle attenuation and skeletal muscle fat content, validated through biopsy specimens, emphasising the accuracy and reliability of CT in detecting muscle fat infiltration [21, 63]. Quantitative analysis based on CT attenuation values allows a reproducible and objective assessment of muscular fat fraction, and it has been particularly effective in predicting clinical outcomes, such as cancer survival and mortality in CKD and cirrhosis [6, 7, 8, 10].

Recently, there have been advances in artificial intelligence (AI) that have allowed for the automatic segmentation of specific muscular areas and the computation of attenuation values to estimate the extent of fat infiltration accurately [64]. Deep learning approaches, particularly convolutional neural networks (CNN), achieve high accuracy for muscle and adipose tissue segmentation, substantially reducing observer variability and analysis time [65]. These methods allow automated body composition analysis from routine clinical CT scans and support large‐scale population studies [9, 66]. Importantly, AI‐enabled volumetric assessments capture the heterogeneous and region‐specific distribution of muscle fat infiltration across multiple muscle groups.

The strengths of CT for IMF measurement are its speed, widespread availability and simplicity. CT scans are routinely performed for various medical conditions, allowing for opportunistic retrospective analysis without additional imaging costs. CTs also benefit from absolute standardised values (HU) that ensure consistency and reproducibility across different scanners when properly calibrated. Additionally, CT has proven exceptionally effective in distinguishing and quantifying IMAT, enabling detailed segmentation and clear differentiation between muscle groups and adipose tissue.

However, CT has several limitations. A drawback of monitoring patients with CT scans is the inherent ionising radiation exposure associated with them. This restricts its repeated use or longitudinal monitoring, particularly in healthy individuals, paediatric populations, and pregnant women [21]. Therefore, CT is often used opportunistically, leveraging scans obtained for other clinical indications. CT is also limited by its reduced sensitivity to detect subtle or early changes in muscle fat infiltration. Small variations in fat fraction may not produce large swings in HU. Early studies have demonstrated that CT is less sensitive than MRI to early or subtle fat infiltration [67]. Two muscles with similar average HU might still differ in the micro‐distribution of fat that MRI could detect. Differences in density may also be influenced by factors beyond fat content. Fluid retention is particularly relevant in conditions such as cardiac, renal, and hepatic failure. Muscles with increased fluid retention will show reduced muscle density, since water has a lower radiological density (0 HU) than that of protein [68]. Muscle glycogen levels could also affect muscle density measurements; although data from glycogen storage disease suggest this, the evidence remains limited [69, 70]. Dixon or spectroscopy techniques may be more accurate in these clinical settings [71]. Furthermore, despite the existence of some validated thresholds, such as radiodensity at the level of L3, < 41 HU in those with a BMI < 25 kg/m^2^ and < 33 HU in those with a BMI ≥ 25 kg/m^2^, there is a lack of consensus across the field [72, 73]. Age, obesity, diabetes and surgical procedures are known to cause reductions in muscle attenuation, making establishing absolute cut‐offs challenging [74].

Despite these issues, CT remains a pragmatic method for large cohorts due to its availability and ease of retrospective analysis. When CT and MRI have been compared, they generally show good agreement in ranking individuals' muscle fat levels, although MRI may detect slight fat inhomogeneities that CT smooths over [75].

### 3.5 Ultrasound

MRI and MRS remain the reference standards for IMF quantification, but their high cost, limited portability and logistical demands restrict routine use. Ultrasound (US) has emerged as a promising, accessible alternative for estimating IMF at the point of care, offering potential value for large‐scale screening, bedside monitoring and use in resource‐limited settings, without the exposure to ionising radiation.

Ultrasonography evaluates IMF indirectly via echogenicity measurements, where increased echogenicity indicates a higher content of intramuscular fat or fibrosis, as fat and fibrous tissue scatter sound waves [76]. Quantitative ultrasound (QUS) measures, like echo intensity, can serve as proxies for myosteatosis. Studies have shown that ultrasound echo intensity correlates with MRI‐estimated intramuscular fat and with muscle performance metrics [77, 78]. In addition, US also allows for the simultaneous measurement of muscular volume, fascicle length and pennation angle through the acquired images, providing a complementary functional context [79].

Despite these advantages, current US techniques face reproducibility challenges. Measurements are sensitive to operator technique, probe pressure, transducer frequency, machine type and gain settings, and these factors can limit comparability across sites [76, 80]. Inter‐device variability is a particular barrier to multicentre trials [76]. Figure 4 demonstrates this inter‐device variability [80].

**FIGURE 4:**
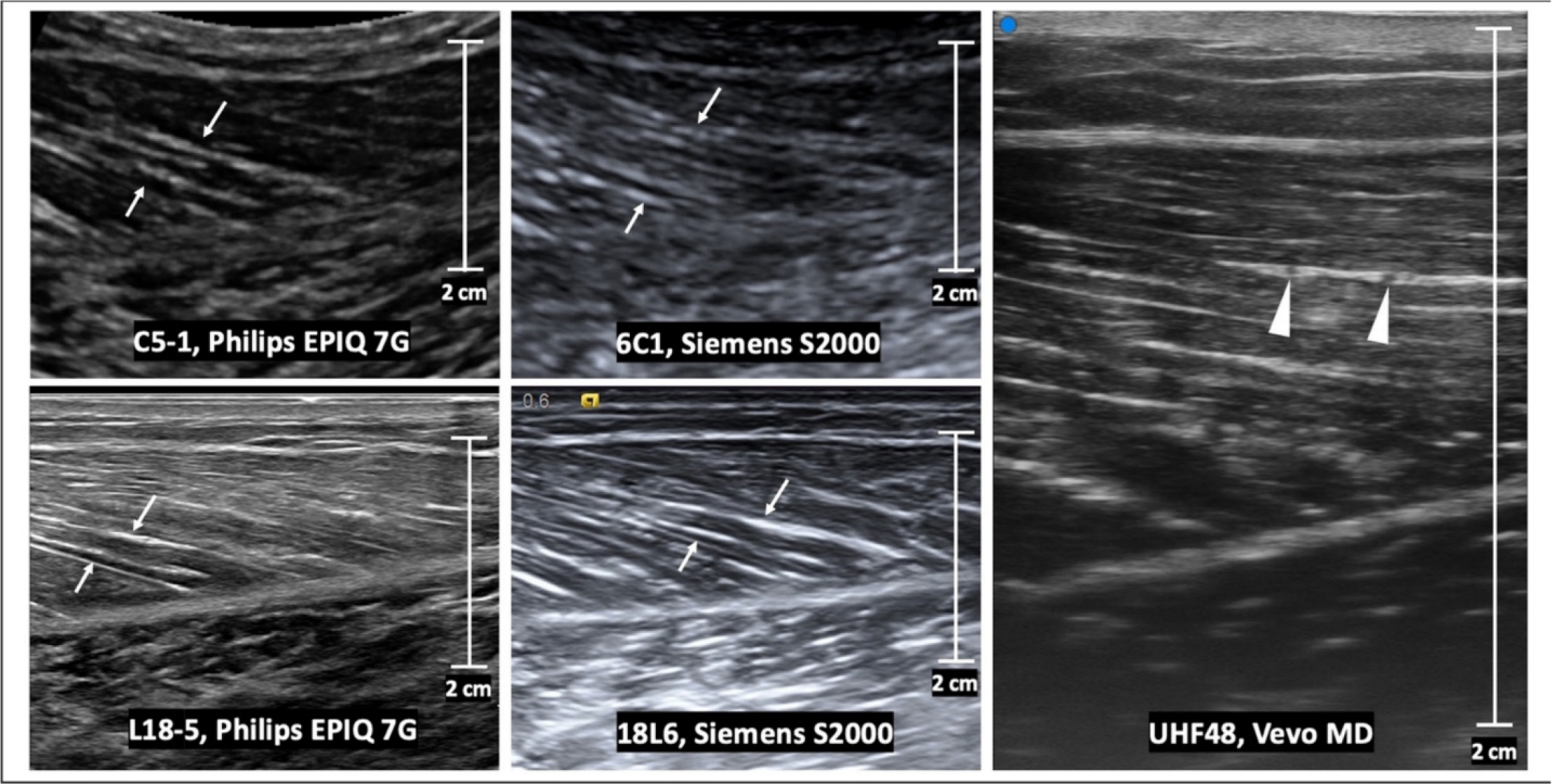
Longitudinal B‐mode ultrasound images of the medial gastrocnemius muscle in a healthy volunteer, acquired with different transducers. Arrows indicate the echogenic perimysia, which are evident on all images but differ in appearance with transducer frequency and vendor reconstruction algorithm. At ultra‐high frequency (UHF48 transducer), arrowheads indicate intramuscular veins within a layer of the perimysium. Scale bars represent 2 cm. The figure was reprinted from a previously published open‐access journal article by Ashir et al. under the terms of the Creative Commons CC BY license [80].

Efforts to improve standardisation are underway. The SARCopenia through UltraSound (SARCUS) consensus has defined anatomical landmarks for 39 muscles, thereby improving acquisition reproducibility, although optimal devices and QUS parameters remain undefined [79]. Separately, the University of Barcelona group has proposed a cadaver‐validated three‐point semi‐quantitative grading system for sarcopenia—ranging from grade 1 (normal) to grade 3 (severe)—which demonstrates excellent inter‐ and intra‐observer reliability and high concordance with histology [81].

US cannot currently distinguish between IMF compartments (IMCL, EMCL, IMAT) or differentiate myosteatosis from myofibrosis, as both fat and fibrosis increase echo intensity [82]. Additionally fluid retention may confound measurements in conditions such as renal, cardiac or hepatic failure, and US is less reliable for deep muscle groups [83]. Advances in automated image analysis, AI‐based segmentation and harmonised acquisition protocols may help overcome these barriers. While US is unlikely to replace MRI for precise IMF quantification, it holds considerable promise as a complementary, portable tool for IMF screening and longitudinal monitoring once methodological consistency is achieved.

## 4 Muscle, Population‐Specific and Activity‐Based Variability in IMF

IMF accumulation is multifactorial, involving age‐related changes in fibro‐adipogenic progenitor cell behaviour, reduced mitochondrial oxidative capacity, hormonal shifts and chronic low‐grade inflammation, with detailed mechanistic reviews available [84]. Here, we focus on the demographic and lifestyle modifiers and their implications for biomarker interpretation.

Establishing IMF as a biomarker requires defining its normal range across populations. MRI fat fraction reference values are emerging; levels are clearly context‐dependent, varying by muscle group, age, sex, body composition, activity level and ethnicity. Belzunce et al. reported ≈8%–10% IMF in gluteus medius and minimus, with no sex difference, whereas gluteus maximus (GMAX) typically contained ≈15% in young males and ≈20% in young females [45]. Thus, even in healthy individuals, muscles exhibit inherently different fat content, likely reflecting functional demands and fibre‐type composition. Dixon MRI studies on the thigh muscles have also demonstrated significant variability across muscles, with some muscles displaying nearly double the fat content of their neighbours [20]. This highlights substantial intra‐individual variability. Weight‐bearing or slow‐twitch dominant muscles tend to accumulate more IMCL than predominantly fast‐twitch muscles [85], underscoring that ‘normal’ levels of IMF are not uniform across muscle groups.

Ageing is a major determinant of IMF. MRI cohort studies show strong positive correlations between age and muscle fat fraction, reinforcing myosteatosis as a contributor to age‐related muscle quality decline [86]. Hogrel et al. used Dixon MRI to compare young adults aged 20–30 years and elderly adults aged 70–80 years, demonstrating noticeable differences [20]. Quadriceps muscles in older men contained four to five times more IMF than those of young men, while older women exhibited two to three times higher IMF than younger women, despite only modest differences in muscle cross‐sectional area. The effect of ageing on muscle fat infiltration was also not uniform across muscle groups; knee extensors and adductors, in particular, show substantial absolute increases in fat infiltration. This progressive fatty infiltration may explain why muscle strength and mobility decline with age beyond what is expected from muscle mass alone [20, 25].

Sex influences IMF, although its effects seem less significant than those of age. In population studies, women generally show slightly higher IMF than men, particularly after menopause, even when BMI‐matched [86, 87]. Belzunce et al. also reported that healthy women in their cohort had about 20.4% IMF in GMAX compared to 14.8% in men, a difference thought to be mediated by hormonal influences and divergent fat distribution patterns [15]. However, a later study with a larger cohort found that sex differences diminished or disappeared when conditioning level was accounted for. In a 2023 MRI study of recreational cyclists, men and women displayed equivalent gluteal IMF, ≈12%, once adjusted for body size and activity, though women still had more subcutaneous fat [46]. This finding suggests that regular exercise can effectively override small inherent sex‐based differences in muscle fat content.

Body composition, especially BMI, further interacts with these factors. There is a moderate positive relationship between BMI and IMF, such that overweight individuals often demonstrate higher IMF values, though with wide inter‐individual variation [86]. Some lean individuals, especially in high‐risk ethnic groups, may still accumulate disproportionately high IMF [88]. This highlights once again that IMF cannot be interpreted in isolation from the broader body composition and demographic context.

Ethnicity introduces an additional layer of complexity. A childhood MRS study showed that South Asian children carry higher levels of intramyocellular lipid than their Malay or Chinese peers, even after adjusting for BMI, indicating early‐life ethnic differences in muscle lipid metabolism [89]. In adults, several studies suggest that African American and Hispanic populations accumulate greater IMF than Caucasians for a given body size, even though Black individuals often have lower visceral adiposity [88, 90]. This paradoxical pattern might explain increased insulin resistance in these groups, despite their relatively favourable visceral fat profiles. Inuit women further exemplify this; CT analysis reveals higher overall fat levels and lower muscle density compared to European women, highlighting population‐specific differences in body composition profiles [91]. Similarly, East Asian populations seem to develop metabolic problems at relatively low IMF levels, making it difficult to use universal cut‐offs. These findings emphasise that normative values derived from European or North American cohorts are not universally applicable, and ethnicity‐specific reference standards will be required.

Lifestyle factors, especially physical activity, exert perhaps the most dynamic influence on IMF. Athletes consistently demonstrate markedly lower extramyocellular fat than sedentary individuals, regardless of sex or age. Inactivity, whether associated with MSK conditions or not, strongly predisposes to myosteatosis [92, 93]. A cross‐sectional Dixon MRI study of the gluteus maximus found that inactive people with hip osteoarthritis had intramuscular fat fractions of about 26%, nearly double the 14% observed in age‐matched healthy but physically active peers [15]. Figure 5 demonstrates observable differences in fat with activity [15]. Exercise has consistently demonstrated effectiveness in preventing or reducing IMAT accumulation in older adults and those with moderate functional impairments, as well as maintaining muscle function and strength in those participating in exercise interventions [19, 94, 95]. Meanwhile, sedentary lifestyles promote fat infiltration even in young adults [96].

**FIGURE 5:**
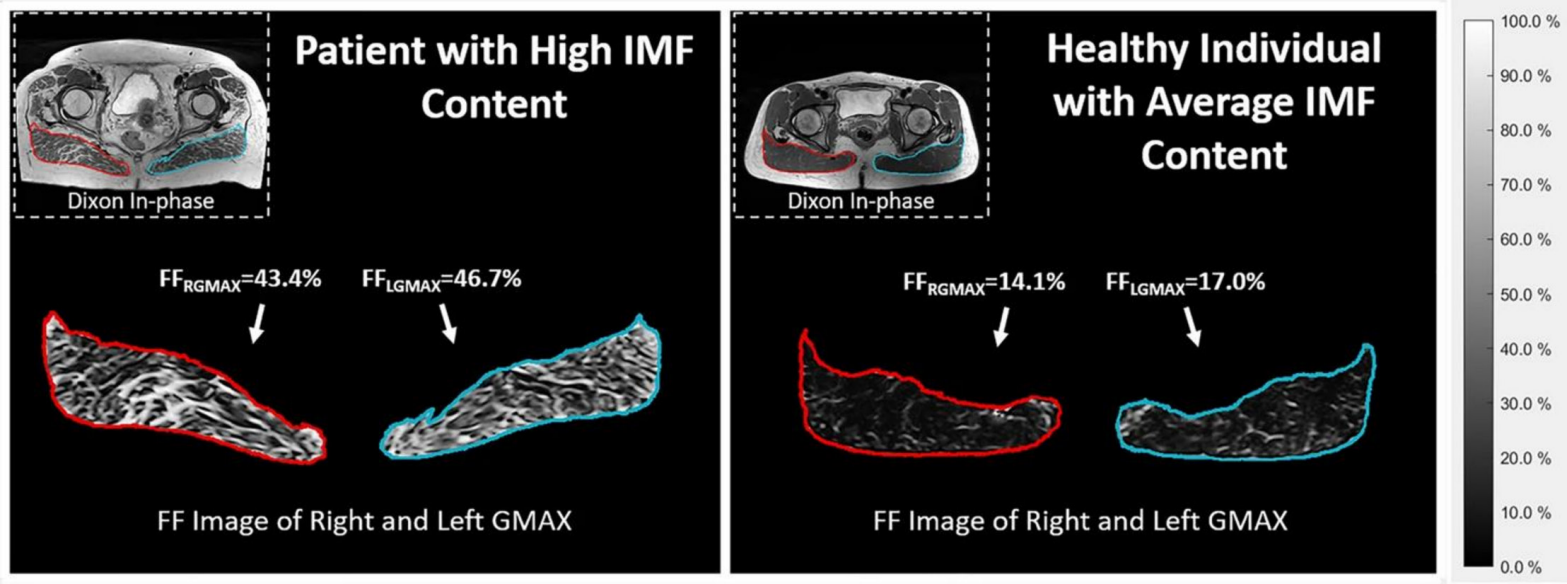
Fat fraction (FF) maps of the gluteus maximus (GMAX) in two individuals: a patient with higher intramuscular fat (IMF) infiltration (left) and a healthy individual from the medium physical activity group (average IMF content) (right). FF maps are shown for the right and left GMAX, with mean values given for each muscle (FF_RGMAX_, right GMAX; FF_LGMAX_, left GMAX). The greyscale bar denotes FF from 0% to 100%. The corresponding Dixon in‐phase image for each case is inset. The figure was reprinted from a previously published open‐access journal article by Belzunce et al. under the terms of the Creative Commons CC BY license [15].

However, endurance training appears to increase IMCL stores alongside mitochondrial density, increasing readily available fuel for prolonged exercise. This is why highly trained athletes can have intramyocellular fat yet remain insulin sensitive, known as the ‘athlete's paradox’ [97, 98]. This leads to a U‐shaped relationship between IMCL and insulin sensitivity: high IMCL in insulin‐sensitive athletes, low IMCL in insulin‐sensitive non‐athletes, and high IMCL in insulin‐resistant states [99, 100]. In athletes, IMCL is stored within oxidative fibres and efficiently utilised during exercise. Lipid droplets in trained muscle are smaller and more closely associated with mitochondria, potentially supporting faster turnover and offering a micro‐environmental explanation for the paradox [101].

Collectively, these findings highlight both the promise and complexity of IMF as a biomarker. ‘Normal’ values depend on muscle group, age, sex, body composition, ethnicity and activity level. Clinical implementation will therefore require standardised imaging protocols alongside population‐specific reference frameworks.

## 5 Responsiveness of IMF to Interventions and Its Role as a Biomarker

IMF is a modifiable tissue depot, and understanding its responsiveness to interventions is central to its value as a biomarker. Evidence from exercise, dietary, and pharmacological interventions demonstrates that IMF can be reduced and that these changes correlate with meaningful improvements in insulin sensitivity, muscle strength, and functional outcomes.

A single bout of endurance exercise can acutely deplete IMCL stores. A recent meta‐analysis of acute exercise studies found that a session of cycling leads to a net ≈24% reduction in IMCL content in exercised muscles, primarily type 1 fibres [102]. This IMCL degradation occurred regardless of whether exercise was done fasted or fed. These findings confirm that muscle fibres utilise internal lipid depots during exercise. The timing of IMF measurement is therefore critical, as post‐exercise MRS may transiently show reduced IMCL due to acute oxidation.

Regular physical activity and structured exercise programs have been shown to prevent age‐related fat accumulation in muscle and even reduce existing fatty infiltration. Goodpaster et al. showed that 12 months of a moderate physical activity program in older adults prevented the increase in thigh muscle fat seen in a sedentary control group, the control group gained ≈18% IMF against only ≈2% in the active group [19]. The exercise group also avoided the strength loss that the controls experienced, despite similar small losses of muscle mass. The results suggest that aerobic exercise programs can halt the progression of muscle fat infiltration that accompanies inactivity and ageing. A 2021 meta‐analysis of 19 randomised controlled trials reported that combined aerobic and resistance training reduces intramuscular adipose infiltration and increases muscle density in at‐risk adults, with the greatest benefits seen when exercise is paired with weight loss [103]. For example, in older women with type 2 diabetes, 6 months of concurrent aerobic and resistance training produced a substantial and statistically significant improvement in thigh muscle attenuation [104]. Similarly, older men undertaking aerobic exercise saw increases in muscle density, especially when exercise was coupled with weight loss [95]. These findings reinforce that exercise can meaningfully improve muscle composition, particularly when multi‐modal programs are employed. Interestingly, exercise without weight loss tends to produce inconsistent changes in IMF from a recent meta‐analysis [105]. This highlights that reductions in muscle fat are most robust when exercise‐induced muscle hypertrophy is accompanied by overall fat loss.

Caloric restriction and bariatric surgery consistently reduce ectopic fat depots, including IMF, and improve muscle density and strength per mass [106, 107]. Combined exercise and hypocaloric diets produce greater reductions than exercise alone [105]. Weight loss reduces systemic inflammation and increases fat availability, which likely facilitates the mobilisation of intermuscular fat stores for energy [1, 108]. However, rapid weight loss risks lean mass loss, underscoring the importance of protective factors such as resistance training and adequate protein intake [106, 107, 109]. In the long term, maintaining a healthy body fat level is essential for keeping IMF low. Multiple studies have demonstrated that higher BMI independently predicts increased muscular fat infiltration, even among those with similar activity levels [15, 45].

Pharmacological therapies have recently shown potential to reduce IMF, particularly glucagon‐like peptide‐1 receptor agonists (GLP‐1 RA), such as liraglutide and semaglutide. In a 2024 randomised trial, liraglutide produced a modest but significant reduction in this IMF and reduced the prevalence of adverse muscle composition compared with placebo [110]. Complementary imaging studies with semaglutide demonstrate a parallel decline in visceral and intramuscular fat [111, 112]. However, similar to bariatric surgery, GLP‐1 RAs also reduce absolute muscle mass alongside fat loss, raising concerns about sarcopenic obesity, particularly in older or frail individuals [112, 113]. The long‐term effects on muscle composition remain unclear, as most studies lack detailed quantitative imaging assessments to characterise changes in muscle quality and IMF distribution over time. Adequate protein intake and resistance exercise may be important for preferentially preserving lean tissue during GLP‐1 RA therapy [112].

Several mechanisms may underpin these effects. GLP‐1 RAs induce negative energy balance, enhancing lipolysis and mobilisation of intramuscular adipose. They improve insulin sensitivity, which promotes oxidation of IMCL, while preclinical studies show increases in oxidative fibre type ratios, mitochondrial content, and muscle protein synthesis [110]. Together, these adaptations support fat clearance and functional preservation. Though currently, much of the research on GLP‐1 RAs does not require assessments of muscle composition, and findings are generally limited and underpowered [112].

Disease‐specific considerations are emerging. In cases of cancer and renal failure, hypoalbuminaemia, an indicator of malnutrition and systemic inflammation and its impact on protein metabolism, has been independently associated with myosteatosis [7, 114]. This highlights the complex interplay between nutritional status, inflammation, and muscle quality in these conditions. In cancer cachexia, multimodal approaches combining resistance exercise with nutritional support show promise. Fish oil enriched with Eicosapentaenoic acid (EPA) appears to reduce intramuscular adipose tissue during chemotherapy, whereas high‐protein supplementation (> 1.5 g/kg/day) may help preserve muscle mass [115, 116]. However, data on IMF‐specific interventions in cancer remain limited; further research is required to establish optimal strategies.

In type‐2 diabetes, combined aerobic and resistance training shows particular efficacy in reducing IMF alongside improvements in insulin sensitivity and glycemic control [104]. In MASLD, the liver‐muscle metabolic axis appears central to pathogenesis. Interventions targeting myosteatosis, including combined exercise and dietary modification, bariatric surgery or hybrid training, may be indirectly hepatoprotective by modulating inter‐organ communication and reducing systemic inflammation [117]. Physical activity reduces both muscle and hepatic fat while enhancing insulin sensitivity.

In summary, IMF appears to be meaningfully malleable, with disease‐specific strategies emerging across medical specialities. Utilising IMF as an intervention biomarker, however, requires careful methodological consideration. Multi‐echo Dixon MRI offers the most validated approach for longitudinal monitoring, owing to its reproducibility and ability to quantify whole muscles. Muscle selection should align with intervention targets. Assessment intervals must be tailored to intervention type and duration, as meaningful structural changes require sustained intervention over months. Interpreting the results also requires adjustment for age, sex, BMI, and disease state, given the previously discussed population variability in normative values. Currently, no consensus thresholds define clinically meaningful change, highlighting the need for standardised protocols and disease‐specific minimal detectable change criteria.

## 6 Open Challenges and Future Directions

Despite the increasing interest in IMF, several challenges must be overcome before it can be accepted as a biomarker of muscle health.

### 6.1 Standardisation of Measurement

A fundamental hurdle is the lack of standardised measurement protocols for quantifying intramuscular fat. Different studies employ MRI, CT or ultrasound, with further heterogeneity within modalities: MRI can involve two‐point or multi‐echo Dixon, and manufacturers have differing protocols for scan acquisition; CT relies on attenuation cut‐offs, and the use of phantoms in clinical practise is not yet standardised to calibrate measurements; and QUS measures echo intensity with variable gain settings. This makes comparisons across cohorts difficult and hampers reproducibility.

For MRI, multi‐echo Dixon PDFF is emerging as the most reliable method, but variability in scanner calibration and sequence design not specific to muscular triglyceride can affect results. Reproducibility of region‐of‐interest/voxel in MRS still limits generalisability. Ultrasound is inherently plagued by operator dependence. Though the SARCUS groups' work on defining anatomical landmarks is an excellent step in improving standardisation in ultrasound. These issues were highlighted in the 2020 National Institutes of Health (NIH) consensus workshop on myosteatosis, which explicitly called for international efforts to harmonise definitions, nomenclature, and protocols across imaging modalities [1]. Such initiatives, akin to the standardisation achieved for bone density scans or hepatic steatosis, will be essential for advancing IMF as a biomarker.

### 6.2 Defining Pathological Thresholds

Unlike osteoporosis or hepatic steatosis, myosteatosis currently lacks universally agreed cut‐offs. The clinical meaning of a given IMF level is strongly context‐dependent: 15% fat in the quadriceps may be benign in an 80‐year‐old but abnormal in a 30‐year‐old athlete. Baseline fat levels also vary across muscles within the same group and between different groups, complicating threshold setting. Some have attempted to derive criteria such as mean density values of less than 41 HU in individuals with a body mass index (BMI) under 25 kg/m^2^ or less than 33 HU in those with a BMI greater than or equal to 25 kg/m^2^ in oncology; however, this is still not standardised [62]. The NIH workshop underscored the need for large, multi‐ethnic longitudinal studies to link IMF with outcomes such as mobility, falls, fractures, diabetes, and mortality.

Ultimately, IMF should follow the model established for grip strength in sarcopenia, where age and sex specific normative reference tables enable identification of low values [118]. Developing analogous reference ranges for IMF across muscles, stratified by age, sex and ethnicity, would provide clinicians with interpretable benchmarks comparable to existing sarcopenia diagnostic criteria. Thresholds may also need to be disease‐specific; IMF levels that predict poor outcomes in cirrhosis or renal failure might differ from those relevant in sarcopenia of ageing. Beyond diagnostic thresholds, minimal detectable change criteria are also required to define clinically meaningful responses to intervention, as monitoring biomarkers need different validation than diagnostic ones.

### 6.3 Context‐Specific Interpretation

Another unresolved issue is how context influences interpretation. The athlete's paradox illustrates this: high intramyocellular lipids can coexist with good metabolic health in endurance athletes. However, the same IMCL levels in sedentary individuals are linked to insulin resistance and frailty. This underlines that IMF cannot be considered a uniform risk factor. A potential solution is composite indices: For instance, a ‘muscle quality index’ that integrates IMF with strength or power measures could differentiate harmful fat infiltration from benign adaptations.

Beyond fat quantification, the index might also consider lipid composition and spatial distribution within skeletal muscle, which are increasingly recognised as biologically relevant. Recent mass spectrometry imaging studies of muscle histology enable high‐resolution mapping of lipid species and their microregional organisation, offering a complementary framework for contextualising MRI‐derived measures of muscle fat infiltration [119, 120].

The 2020 NIH consensus workshop highlighted this complexity, emphasising that IMF represents multiple compartments and lipid species that imaging alone may not fully resolve. Future work should explore whether droplet size, lipid saturation, or deposition pattern correlates with specific outcomes. Clear interpretive frameworks will be critical for preventing over‐ or underdiagnosis when IMF is reported clinically.

### 6.4 Advanced Imaging and AI

AI offers an effective way to overcome the scalability limitations that have traditionally restricted the study of intramuscular fat. Automated segmentation and quantification enable high‐throughput analysis of muscle composition from CT and MRI datasets, including routine clinical scans, which would otherwise be too time‐consuming to process manually. This enables population‐level research and creates opportunities to identify distinct patterns of fat infiltration across different muscle groups that may be relevant to frailty, metabolic conditions, and disease‐related risks.

Importantly, volumetric and multi‐muscle analyses enable assessment of the spatial variability of IMF, an aspect poorly captured by single‐slice or region‐based methods. This is especially relevant in longitudinal and interventional studies, where regional changes in fat infiltration may happen before observable changes in muscle mass or strength.

Meanwhile, progress in quantitative MRI, including techniques that combine fat fraction with markers of fibrosis, inflammation, or lipid compartmentalisation, may allow more comprehensive assessments of muscle health. Overall, these developments promote a move towards a multidimensional characterisation of muscle composition.

## 7 Conclusion

Intramuscular fat has emerged as a key determinant of muscle quality, complementing traditional metrics such as muscle mass and strength. The evidence, with exceptions, links higher IMF with poorer muscle function, greater frailty, metabolic dysfunction, and adverse surgical outcomes. Yet the interpretation of IMF is nuanced; what is pathological in one context may be benign in another. The review has outlined how new quantitative IMF assessments could serve as a biomarker of muscle health. They provide information on previously qualitative aspects of muscle health that are otherwise invisible to volume‐based assessments, thereby capturing the ‘hidden’ dimension of sarcopenia.

At the same time, significant barriers remain. Standardisation is still lacking, despite ongoing efforts by multiple research groups. Normative reference ranges are incomplete, and clinically actionable thresholds are not established. IMF exhibits diversity in its biology and function; it comprises IMCL and EMCL, which have distinct roles, and its effects vary with age, sex, activity and ethnicity. Our understanding of the complexity of the issue has led to IMF being recognised as a distinct but related component of skeletal muscle deficit. This framing emphasises that IMF should be studied not in isolation but in conjunction with strength and performance measures.

The challenges ahead centre on integrating IMF into a multidimensional concept of muscle health. Setting thresholds through longitudinal, multi‐ethnic cohorts will be essential. Advances in imaging protocols, AI‐driven analysis, large imaging databases, and mechanistic studies will improve measurement and interpretation. Ultimately, IMF's potential is to personalise care by identifying older adults with high IMF who might benefit from targeted exercise or nutrition, or by tracking whether an intervention genuinely enhances muscle quality rather than just mass. If hurdles are addressed, IMF could become part of routine clinical assessment, much like bone density or liver fat, enabling clinicians to better stratify risk across conditions ranging from sarcopenia to cirrhosis to cancer cachexia, monitor response to therapy, and preserve function across a lifespan.

## Funding

This research study was funded by the Arthroplasty for Arthritis Charity, the Trustees of the London Clinic Charity, the Maurice Hatter Foundation, the RNOH Charity, the Rosetrees Trust, the Cleveland Clinic London Foundation and the Stoneygate Trust and supported by researchers at the National Institute for Health Research, University College London Hospitals Biomedical Research Centre.

## Conflicts of Interest

The authors declare no conflicts of interest.

## Study Design

Narrative review.
